# Correction: Bayesian inference and comparison of stochastic transcription elongation models

**DOI:** 10.1371/journal.pcbi.1009314

**Published:** 2021-08-11

**Authors:** The PLOS Computational Biology Staff

There are a number of errors in the caption for S1 Fig. The publisher apologizes for the errors. Please see the correct S1 Fig caption here.

## Supporting information

S1 FigSimulations of the elongation pathway.Each point is a single simulation of the full *rpoB* gene (4029 nt). Parameters on the x- and y-axis are sampled uniformly at random from the displayed range at the beginning of each trial. The z-axis of each plot (mean elongation velocity) is then measured from the respective simulation. [NTP] and *F* held constant at 1000 *μ*M and 0 pN respectively. (A): Relationship between $\Delta G_{\tau }^{‡}$ and *k*_*cat*_ with binding at equilibrium (Model 8). (B) Relationship between *k*_*bind*_ and *k*_*cat*_ for the kinetic binding model with translocation at equilibrium (Model 2). (C) Relationship between *K*_*D*_ and *k*_*bind*_ with translocation held at equilibrium (Model 2). *K*_*D*_ and *k*_*bind*_ sampled uniformly from specified range and velocity is measured. Samples with simulated velocities outside of the range 0.1–0.2 bp/s were discarded. [NTP] = 10 *μ*M and *k*_*cat*_ = 100 s^−1^.(PDF)
