# Supplementary material for: Bayesian inference and comparison of stochastic transcription elongation models
Source: PLoS Comput Biol. 2020 Feb 14;16(2):e1006717. doi: 10.1371/journal.pcbi.1006717 (PMC7046298; doi:10.1371/journal.pcbi.1006717)
Supplement: S1 Appendix — The Gillespie algorithm is described. (PDF) [file pcbi.1006717.s001.pdf]

S1 Appendix: Stochastic simulation

Reactions are simulated using the Gillespie algorithm [1]. Given the current state s and a set of possible reactions $s \to s_1, s \to s_2, \ldots, s \to s_n$ with rate constants $k_1, k_2, \ldots, k_n$, the next reaction to perform is sampled proportional to its rate:

$$p(s \to s_i) = \frac{k_i}{\sum\limits_{j=1}^{n} k_j}. \tag{1}$$

The amount of time the reaction takes to occur is sampled from the exponential distribution with rate $\sum\limits_{j=1}^{n} k_j$.

References

[1] Gillespie DT. Exact stochastic simulation of coupled chemical reactions. The journal of physical chemistry. 1977;81(25):2340–2361.
