# Supplementary material for: Bayesian inference and comparison of stochastic transcription elongation models
Source: PLoS Comput Biol. 2020 Feb 14;16(2):e1006717. doi: 10.1371/journal.pcbi.1006717 (PMC7046298; doi:10.1371/journal.pcbi.1006717)
Supplement: S2 Appendix — Master equations for the four equilibrium model variants are presented. (PDF) [file pcbi.1006717.s002.pdf]

S2 Appendix: Chemical master equations

The chemical master equations for the four equilibrium variants (Fig 4A) of single nucleotide addition cycles are provided in this section. Transcription of a full gene involves chaining multiple of these single-cycle models together.

Translocation and binding equilibrium model

The state pathway of a single-cycle of the translocation and binding equilibrium model is

$$S(l,0) \underset{K_\tau(l)}{\rightleftharpoons} S(l,1) \underset{K_D}{\rightleftharpoons} S_N(l,1) \underset{k_{cat}}{\longrightarrow} S(l+1,0) \tag{1}$$

where S_N denotes a state where NTP is bound. As states $S(l,0)$, $S(l,1)$, and $S_N(l,1)$ are in mutual equilibrium they can be coalesced into one state. Let $S(l)$ be the coalesced state that exists in equilibrium between the three, then the pathway may be rewritten as

$$S(l) \underset{\omega_{cat}(l)}{\longrightarrow} S(l+1,0) \tag{2}$$

where $\omega_{cat}(l)$ is the *effective* rate of catalysis from this state. This term is equal to k_{cat} multiplied by the proportion of time that the coalesced state has NTP bound, ie. in state $S_N(l,1)$.

$$\omega_{cat}(l) = p(S_N(l,1))\ k_{cat} \tag{3}$$

$$= \frac{\frac{[NTP]}{K_D}}{1 + \frac{[NTP]}{K_D} + exp\{-(\Delta G^{(bp)}_{S(l,0)} - \Delta G^{(bp)}_{S(l,1)} - \Delta G_{\tau 1})\}}\ k_{cat} \tag{4}$$

Let $p(S,\mathbb{T})$ be the probability of the system existing in state S at time $\mathbb{T}$. Under this model, the chemical master equation of a single-cycle is:

$$\begin{pmatrix} \frac{dp(S(l),\mathbb{T})}{d\mathbb{T}} \\ \frac{dp(S(l+1,0),\mathbb{T})}{d\mathbb{T}} \end{pmatrix} = \begin{pmatrix} -\omega_{cat}(l) & 0 \\ \omega_{cat}(l) & 0 \end{pmatrix} \begin{pmatrix} p(S(l),\mathbb{T}) \\ p(S(l+1,0),\mathbb{T}) \end{pmatrix} \tag{5}$$

This system is simple enough to solve analytically. Let $p(\mathbb{T})$ be the Markov transition matrix after time $\mathbb{T}$ and let Q be the Markov process transition rate matrix. In these matrices, entry i, j is the probability (p) or rate (Q) of transition from i to j.

$$
\begin{aligned}
p(\mathbb{T}) =& exp\{Q\mathbb{T}\} && (6)\\
=& exp\left\{ \begin{pmatrix} -\omega_{cat}(l) & \omega_{cat}(l) \\ 0 & 0 \end{pmatrix} \mathbb{T} \right\} && (7)\\
=& \begin{pmatrix} e^{-\omega_{cat}(l)\mathbb{T}} & 1 - e^{-\omega_{cat}(l)\mathbb{T}} \\ 0 & 1 \end{pmatrix} && (8)
\end{aligned}
$$

It is noted that as the time $\mathbb{T}$ approaches infinity, the probability of the system existing in state $S(l+1, 0)$ approaches 1, because it is an absorbing state.

Let $f(\mathbb{T})$ be the probability density of taking *exactly* $\mathbb{T}$ units of time to arrive at state $S(l+1, 0)$, starting from $S(l)$. Because $S(l+1, 0)$ is an absorbing state, computing $f(\mathbb{T})$ is trivial.

$$
\begin{aligned}
f(\mathbb{T}) =& \frac{d}{d\mathbb{T}} \, (1 - e^{-\omega_{cat}(l)\mathbb{T}}) && (9)\\
=& \omega_{cat}(l) \; e^{-\omega_{cat}(l)\mathbb{T}} && (10)
\end{aligned}
$$

Transcribing the full gene requires the traversal of all $L - l_0 + 1$ states. The size of the transition rate matrix for transcribing the entire sequence therefore grows with the length of the DNA sequence.

$$
Q = \begin{pmatrix}
-\omega_{cat}(l_0) & \omega_{cat}(l_0) & 0 & 0 & \dots & 0 \\
0 & -\omega_{cat}(l_0+1) & \omega_{cat}(l_0+1) & 0 & \dots & 0 \\
\vdots & \ddots & \ddots & \ddots & \ddots & \vdots \\
0 & 0 & 0 & 0 & -\omega_{cat}(L-1) & \omega_{cat}(L-1) \\
0 & 0 & 0 & 0 & 0 & 0
\end{pmatrix} \tag{11}
$$

Applying the matrix exponential function to a linear pathway, such as the one described above, has an analytical solution [1]. However, for non-linear systems with an arbitrary number of states (eg. the other 3 equilibrium model variants), analytical solutions may not exist and numerical solutions likely exhibit numerical instabilities [2]. This option was not further investigated in this project (simulation was used instead). However, analytical or stable numerical solutions for $f(\mathbb{T})$ for an arbitrary model would facilitate the use of likelihood functions in Bayesian inference, thereby rendering simulation obsolete.

Binding equilibrium model

The state pathway of a single-cycle of the binding equilibrium model is

$$
S(l,0) \underset{k_{bck(l)}}{\overset{k_{fwd(l)}}{\rightleftarrows}} S(l,1) \underset{K_D}{\rightleftharpoons} S_N(l,1) \xrightarrow[k_{cat}]{} S(l+1,0) \tag{12}
$$

The binding equilibrium assumption permits the coalescence of $S(l,1)$ and $S_N(l,1)$ into a single state $S_N(l)$. Thus, the pathway can be rewritten as

$$
S(l,0) \underset{\omega_{bck(l)}}{\overset{k_{fwd(l)}}{\rightleftarrows}} S_N(l) \xrightarrow[\omega_{cat}]{} S(l+1,0) \tag{13}
$$

where $\omega_{bck(l)}$ is the effective rate of backwards translocation from $S_N(l)$, and ω_{cat} is the effective rate of catalysis. These rates are derived by multiplying their composite rates – $k_{bck(l)}$ and k_{cat} – by the probability of the system existing in the required state to apply the reaction – $p(S(l,1))$ and $p(S_N(l,1))$.

$$\omega_{bck}(l) = p(S(l,1))\ k_{bck}(l) \tag{14}$$
$$= \frac{1}{1+\frac{[NTP]}{K_D}}\ k_{bck}(l) \tag{15}$$

$$\omega_{cat}(l) = p(S_N(l,1))\ k_{cat} \tag{16}$$
$$= \frac{\frac{[NTP]}{K_D}}{1+\frac{[NTP]}{K_D}}\ k_{cat} \tag{17}$$

Let $p(S,\mathbb{T})$ be the probability of the system being at state S at time $\mathbb{T}$. Under this model, the chemical master equation of a single-cycle is:

$$\begin{pmatrix} \frac{dp(S(l,0),\mathbb{T})}{d\mathbb{T}} \\ \frac{dp(S_N(l),\mathbb{T})}{d\mathbb{T}} \\ \frac{dp(S(l+1,0),\mathbb{T})}{d\mathbb{T}} \end{pmatrix} = \begin{pmatrix} -k_{fwd(l)} & \omega_{bck}(l) & 0 \\ k_{fwd(l)} & -\omega_{bck}(l) - \omega_{cat}(l) & 0 \\ 0 & \omega_{cat}(l) & 0 \end{pmatrix} \begin{pmatrix} p(S(l,0),\mathbb{T}) \\ p(S_N(l),\mathbb{T}) \\ p(S(l+1,0),\mathbb{T}) \end{pmatrix} \tag{18}$$

Translocation equilibrium model

The state pathway of a single-cycle of the translocation equilibrium model is

$$S(l,0) \underset{K_\tau(l)}{\rightleftharpoons} S(l,1) \underset{k_{rel}}{\overset{k_{bind}[NTP]}{\rightleftarrows}} S_N(l,1) \underset{k_{cat}}{\longrightarrow} S(l+1,0) \tag{19}$$

The translocation equilibrium assumption permits the coalescence of $S(l,0)$ and $S(l,1)$ into a single state $S_\tau(l)$. Thus, the pathway can be rewritten as

$$S_\tau(l) \underset{k_{rel}}{\overset{\omega_{bind}[NTP]}{\rightleftarrows}} S_N(l,1) \underset{k_{cat}}{\longrightarrow} S(l+1,0) \tag{20}$$

where ω_{bind} is the effective rate of NTP binding.

$$\omega_{bind} = p(S(l,1))\ k_{bind} \tag{21}$$
$$= \frac{exp\{-(\Delta G^{(bp)}_{S(l,1)} + \Delta G_{\tau 1})\}}{exp\{-(\Delta G^{(bp)}_{S(l,1)} + \Delta G_{\tau 1})\} + exp\{-\Delta G^{(bp)}_{S(l,0)}\}}\ k_{bind} \tag{22}$$

Let $p(S, \mathbb{T})$ be the probability of the system being at state S at time $\mathbb{T}$. Under this model, the chemical master equation of a single-cycle is:

$$\begin{pmatrix} \frac{dp(S_\tau(l),\mathbb{T})}{d\mathbb{T}} \\ \frac{dp(S_N(l,1),\mathbb{T})}{d\mathbb{T}} \\ \frac{dp(S(l+1,0),\mathbb{T})}{d\mathbb{T}} \end{pmatrix} = \begin{pmatrix} -\omega_{bind}[NTP] & k_{rel} & 0 \\ \omega_{bind}[NTP] & -k_{rel} - k_{cat} & 0 \\ 0 & k_{cat} & 0 \end{pmatrix} \begin{pmatrix} p(S_\tau(l),\mathbb{T}) \\ p(S_N(l,1),\mathbb{T}) \\ p(S(l+1,0),\mathbb{T}) \end{pmatrix} \tag{23}$$

Full kinetic model

The state pathway of a single-cycle of the full kinetic model is

$$S(l,0) \underset{k_{bck}(l)}{\overset{k_{fwd}(l)}{\rightleftarrows}} S(l,1) \underset{k_{rel}}{\overset{k_{bind}[NTP]}{\rightleftarrows}} S_N(l,1) \xrightarrow[k_{cat}]{} S(l+1,0) \tag{24}$$

Let $p(S, \mathbb{T})$ be the probability of the system being at state S at time $\mathbb{T}$. Under this model, the chemical master equation of a single-cycle is:

$$\begin{pmatrix} \frac{dp(S(l,0),\mathbb{T})}{d\mathbb{T}} \\ \frac{dp(S(l,1),\mathbb{T})}{d\mathbb{T}} \\ \frac{dp(S_N(l,1),\mathbb{T})}{d\mathbb{T}} \\ \frac{dp(S(l+1,0),\mathbb{T})}{d\mathbb{T}} \end{pmatrix} = \begin{pmatrix} -k_{fwd}(l) & k_{bck}(l) & 0 & 0 \\ k_{fwd}(l) & -k_{bck}(l) - k_{bind}[NTP] & k_{rel} & 0 \\ 0 & k_{bind}[NTP] & -k_{rel} - k_{cat} & 0 \\ 0 & 0 & k_{cat} & 0 \end{pmatrix} \begin{pmatrix} p(S(l,0),\mathbb{T}) \\ p(S(l,1),\mathbb{T}) \\ p(S_N(l,1),\mathbb{T}) \\ p(S(l+1,0),\mathbb{T}) \end{pmatrix} \tag{25}$$

References

[1] Jahnke T, Huisinga W. Solving the chemical master equation for monomolecular reaction systems analytically. Journal of mathematical biology. 2007;54(1):1–26.

[2] Moler C, Van Loan C. Nineteen dubious ways to compute the exponential of a matrix, twenty-five years later. SIAM review. 2003;45(1):3–49.
