# Supplementary material for: Bayesian inference and comparison of stochastic transcription elongation models
Source: PLoS Comput Biol. 2020 Feb 14;16(2):e1006717. doi: 10.1371/journal.pcbi.1006717 (PMC7046298; doi:10.1371/journal.pcbi.1006717)
Supplement: S3 Appendix — A description of the MCMC-ABC algorithm and how it is used to infer parameters and models from experimental data. (PDF) [file pcbi.1006717.s003.pdf]

S3 Appendix: MCMC-ABC

Given model/parameters Θ and observed data $D = (D_1, D_2, \dots, D_n)$, Bayesian inference conventionally involves approximating the posterior probability distribution $P(\Theta|D)$ using the likelihood $P(D|\Theta)$ and the prior $P(\Theta)$.

$$P(\Theta|D) \propto P(D|\Theta)P(\Theta). \tag{1}$$

As there is no easily computed likelihood function, simulation is used. The chi-squared test statistic X^2 evaluates how well a given set of parameters fits the data.

$$X^2 = \sum_i \frac{(S_i - D_i)^2}{S_i} \tag{2}$$

where S_i is the mean velocity simulated under the same [NTP] and applied force F that D_i was measured under. The probability that $X^2 = 0$ is equal to the likelihood $P(D|\Theta)$, however it is computationally impractical to only accept parameters into the posterior distribution when the simulation yields $X^2 = 0$. Therefore a threshold ϵ is used and sample Θ_i is accepted into the posterior only if $X^2(\Theta_i) \leq \epsilon$. This method is called approximate Bayesian computation [1, 2]. This is coupled with Markov chain Monte Carlo (MCMC) to give the MCMC-ABC algorithm which is becoming increasingly popular among computational biologists [1, 3].

Each MCMC chain estimated six parameters and the model indicator M. This means the 12 models share the same parameter objects in the MCMC. There are therefore seven terms to estimate: M, k_{cat}, $\frac{k_{rel}}{k_{bind}}$, k_{bind}, $\Delta G_{\tau 1}$, $\Delta G_{\tau}^{\ddagger}$, and δ_1.

When the current model M_i does not use a parameter (eg. in Model 5 δ_1 is not used), the parameter is still estimated even though it is not being used. When the model requires a parameter to be held constant (eg. in Model 1 $\Delta G_{\tau 1} = 0$), the parameter is set to its constant during the simulation. This is done without affecting or being affected by its current estimate, which is used by other models.

To achieve convergence, we used an exponential cooling scheme on ϵ [3] where $\epsilon_{i+1} = \max(\epsilon_{min}, \epsilon_i \gamma)$ for manually tuned values of $0 < \gamma < 1$ and ϵ_0.

Chains which failed to converge were discarded. A heavy-tailed distribution [4] is used as a proposal function, and the parameter to change at each step in the MCMC is selected uniformly at random.

We ran one or more independent MCMC-ABC chains for each selected ϵ_{min} / RNA polymerase combination. Selecting the threshold ϵ_{min} is a critical process in approximate Bayesian computation. Threshold ϵ_{min} must be large enough to achieve convergence within finite computational resources, but small enough that the resulting posterior distribution is still an accurate approximation of the true posterior distribution. For each RNA polymerase we set ϵ_{min} to some initial guess. Then we ran the MCMC chain until the ESS for X^2 was large (> 300) and lowered ϵ_{min} to the bottom 0.05 quantile of the posterior distribution of X^2. This step was repeated until either: a) the distribution of model indicators M converged (model posterior probabilities have changed by less than 0.01, on average). Or, b) the acceptance rate was less than 5%. The values of ϵ_{min} used in the final posterior distributions were 2.39 for RNAP, 0.705 for pol II, and 4.63 for T7 pol (Table 2).

Parameter point estimates (Fig 5) are the geometric median: that is the value which minimises the total Euclidean distance from the other posterior samples. Parameters were normalised into z-scores first. Our code is open source and available at http://www.polymerase.nz. Textfiles containing the posterior distributions and simulation settings are available to download or visualise with the software.

References

[1] Beaumont MA. Approximate Bayesian computation in evolution and ecology. Annual review of ecology, evolution, and systematics. 2010;41:379–406.

[2] Csilléry K, Blum MG, Gaggiotti OE, François O. Approximate Bayesian computation (ABC) in practice. Trends in ecology & evolution. 2010;25(7):410–418.

[3] Ratmann O, Jørgensen O, Hinkley T, Stumpf M, Richardson S, Wiuf C. Using likelihood-free inference to compare evolutionary dynamics of the protein networks of H. pylori and P. falciparum. PLoS Computational Biology. 2007;3(11):e230.

[4] Brewer BJ, Foreman-Mackey D. DNest4: Diffusive Nested Sampling in C++ and Python. arXiv preprint arXiv:160603757. 2016;.
