# Supplementary material for: Bayesian inference and comparison of stochastic transcription elongation models
Source: PLoS Comput Biol. 2020 Feb 14;16(2):e1006717. doi: 10.1371/journal.pcbi.1006717 (PMC7046298; doi:10.1371/journal.pcbi.1006717)
Supplement: S4 Appendix — Simulation-based justifications behind prior distributions for ΔGτ‡, kbind, and krelkbind. (PDF) [file pcbi.1006717.s004.pdf]

S4 Appendix: Prior distributions

Prior for $\Delta G_\tau^\ddagger$, which governs the rates of translocation

RNAP/pol II: to select a prior for $\Delta G_\tau^\ddagger$ we simulated transcription on the *rpoB* gene under Model 3 – the simplest binding equilibrium model. $\Delta G_\tau^\ddagger$ and k_{cat} were sampled uniformly from a relevant range, with K_D held constant at 100 μM and [NTP] = 1000 μM. For each simulation, the mean elongation velocity was calculated. The results are displayed in S1 Fig.

This plot shows that as the energy barrier of translocation ($\Delta G_\tau^\ddagger$) increases, the velocity decreases. If $\Delta G_\tau^\ddagger \gtrsim 8\ k_BT$ then it becomes impossible to achieve a realistic mean velocity, providing a relatively clear upper bound on this parameter. If $\Delta G_\tau^\ddagger \lesssim 3\ k_BT$ then translocation becomes very rapid and the same distribution of velocities is obtained in simulations, irrespective of the exact value of $\Delta G_\tau^\ddagger$. In this case catalysis becomes strongly rate-limiting, and it would be appropriate to apply a partial equilibrium approximation to the translocation step. This provides an effective lower bound for parameter $\Delta G_\tau^\ddagger$. Therefore we centered our prior distribution for $\Delta G_\tau^\ddagger$ in this interval (a normal distribution with a mean of 5.5 and a standard deviation of 0.97, so that the central 99% interval is (3, 8)).

T7 pol: the same analysis was performed, however with $\Delta G_\tau^\ddagger$ at its prior mean of $-3.3\ k_BT$ (S1 Fig).

Prior for k_{bind}, which governs the rate of NTP binding

To select a prior for k_{bind} we performed similar simulations, but instead used Model 2 – the simplest kinetic binding model. k_{bind} and k_{cat} were sampled uniformly from relevant ranges, K_D was set to 100 μM and [NTP] = 1000 μM. (S1 Fig).

Depending on the exact value of k_{cat}, if $k_{bind} \lesssim 0.1\ \mu\text{M}^{-1}\ \text{s}^{-1}$, then it is impossible to achieve a realistic velocity, providing a relatively clear lower bound on this parameter. If $k_{bind} \gtrsim 5\ \mu\text{M}^{-1}\ \text{s}^{-1}$ then binding becomes very rapid and the same distribution of velocities is obtained in simulations, irrespective of the exact value of k_{bind}. Again this is because catalysis becomes strongly rate limiting in this region, and it would be appropriate to apply a partial equilibrium approximation to the binding step. Hence we centered our (lognormal) prior around the interval (0.01, 5) – the conservatively selected bounds reflecting that the experimental data has been collected at differing

NTP concentrations, altering the rate. Performing the same analysis with different parameters gave us a similar prior.

Prior distribution related to rate of NTP release

A model is non-identifiable if two or more parameterisations can produce the same output. Our preliminary results suggested non-identifiability between $\frac{k_{rel}}{k_{bind}}$ and k_{bind} (S1 Fig). When k_{bind} is low (and hence binding is rate-limiting), there is an approximately linear relationship between $\frac{k_{rel}}{k_{bind}}$ and k_{bind}. As k_{bind} increases from 0, the dissociation constant $\frac{k_{rel}}{k_{bind}}$ must also increase in order for the system to achieve the same velocity. However, as binding comes closer to achieving equilibrium, $\frac{k_{rel}}{k_{bind}}$ converges. Most previous estimates of K_D have assumed binding to be at equilibrium. This assumption restrains the values which K_D may take, and subsequently estimates for K_D are typically in the order of 10^1 - 10^2 μM. However for a model in which binding is slow it is expected that estimates of $\frac{k_{rel}}{k_{bind}}$ can be lower. This has indeed been demonstrated by Mejia et al. 2015 [1] who estimated $\frac{k_{rel}}{k_{bind}}$ to be 0.6 μM. Therefore the prior distribution for $\frac{k_{rel}}{k_{bind}}$ must permit both of these binding models to be tested fairly during Bayesian inference. We centered our lognormal prior for $\frac{k_{rel}}{k_{bind}}$ around a very broad range, with a central 95% interval of (0.2, 200).

It is noted that selecting a prior distribution which does not discriminate between the kinetic and equilibrium binding models *a priori* may not be plausible.

References

[1] Mejia YX, Nudler E, Bustamante C. Trigger loop folding determines transcription rate of Escherichia coli's RNA polymerase. Proceedings of the National Academy of Sciences. 2015;112(3):743–748.
