# Supplementary figures and images for: Bayesian inference and comparison of stochastic transcription elongation models
Source: PLoS Comput Biol. 2020 Feb 14;16(2):e1006717. doi: 10.1371/journal.pcbi.1006717 (PMC7046298; doi:10.1371/journal.pcbi.1006717)

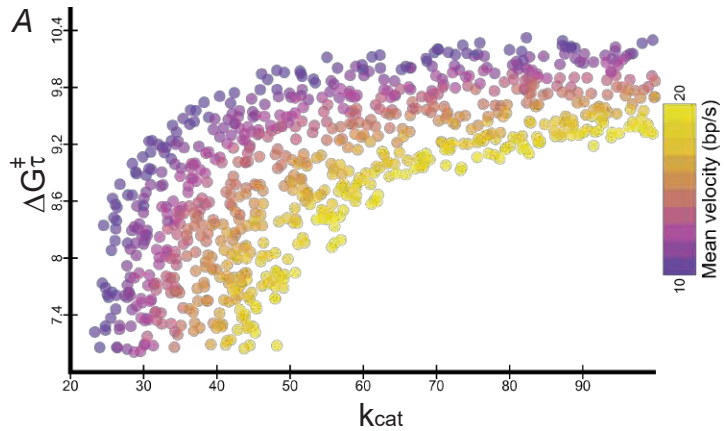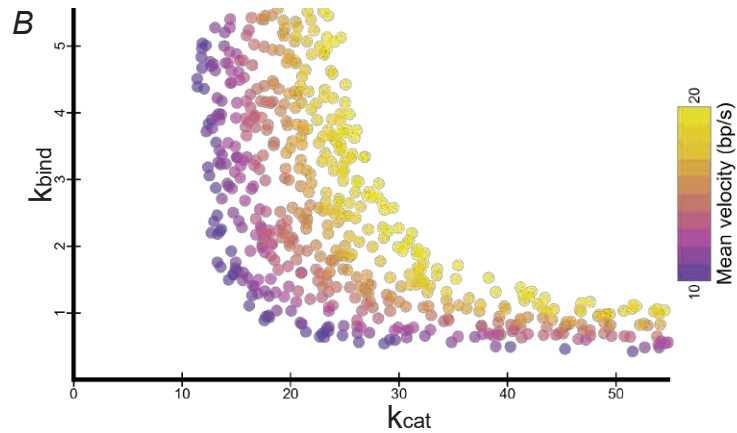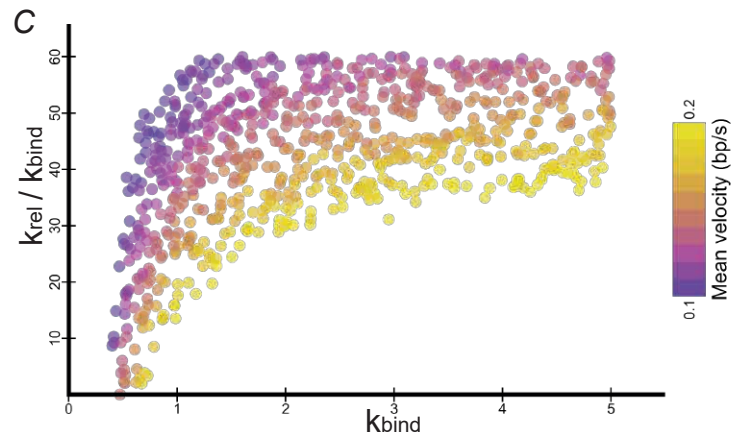

Supplement: S1 Fig — Each point is a single simulation of the full rpoB gene (4029 nt). For (A-C), Parameters on the x- and z-axis are sampled uniformly at random from the displayed range at the beginning of each trial. The y-axis of each plot (mean elongation velocity) is then measured from the respective simulation. [NTP] and F held constant at 1000 μM and 0 pN respectively. (A) and (B): Relationship between ΔGτ‡ and kcat for the melting model with binding at equilibrium (Model 8). ΔGτ1 set to its prior mean (0 for RNAP and pol II, and -3.3 for T7 pol). (C) Relationship between kbind and kcat for the kinetic binding model with translocation at equilibrium (Model 2). (D) Relationship between KD and kbind with translocation held at equilibrium (Model 2). KD and kbind sampled uniformly from specified range and velocity is measured. Samples with simulated velocities outside of the range 1-2 bp/s were discarded. [NTP] = 10 μM and kcat = 100 s−1. (PDF) [file pcbi.1006717.s005.pdf]
